# Quarterly Report of Proceedings

**Published:** 1858-04

**Authors:** 


### QUARTERLY REPORT OF THE PROCEEDINGS.

*Monday, January 4th,* 1858.—A paper was read by Dr. McWilliam "On the Causes which influence the Arrest or Spread of Cholera." By F. H. Johnson, Esq., of Bishopswearmouth. The paper was discussed by Dr. W. Lewis, Mr. Spencer Wells, Dr. Camps, Dr. Snow, and Dr. Mc William.

*Monday, February 1st.*—"On the Influence of Drainage and Water-Supply on Public Health." By John Snow, M.D. A discussion followed, in which Mr. E. Chadwick, Dr. Greenhow, Dr. Milroy, and Dr. Sayer took part.

*Monday, March 1st.*—"On the Investigation of Epidemic Diseases by Experiment." By B. W. Richardson, M D. This paper was discussed by Dr. Greenhow, Dr. Camps, Dr. De Lima, Dr. J. Bird, and Dr. McWilliam.

### MEMBERS ELECTED DURING THE QUARTER.

*Resident.* John Dixon, M.D., Prospect Row, Bermondsey.

*Non-Resident.* Frederick James Brown, M.D., Rome Lane, Chatham, Kent.

